# Occupational Assessments of Risk Factors for Cardiovascular Diseases in Labors: An Application of Metabolic Syndrome Scoring Index

**DOI:** 10.3390/ijerph17207539

**Published:** 2020-10-16

**Authors:** Ching-Yuan Lin, Chih-Ming Lin

**Affiliations:** 1 Department of Laboratory Medicine, Ten-Chan General Hospital, Chung Li, Taoyuan 320, Taiwan; lab02@tcmg.com.tw; 2 Department of Healthcare Information and Management, Ming Chuan University, Taoyuan 333, Taiwan

**Keywords:** metabolic syndrome, labor health examination, severity score, occupational risk

## Abstract

Unlike a traditional diagnosis of metabolic syndrome (MS), a numerical MS index can present individual fluctuations of health status over time. This study aimed to explore its value in the application of occupational health. Using a database of physiological and biochemical tests and questionnaires, data were collected from 7232 participants aged 20 to 64 years who received occupational health screenings at a health screening institution in 2018. Using confirmatory factor analysis, five components of MS were used to design an MS severity scoring index, which was then used to evaluate the risks of occupation factors. Waist circumference was the largest loading factor compared with the other MS components. Participants who worked in the traditional industrial, food processing, or electronic technology industries had higher MS severity than those in the logistics industry. Those who worked as a manager or over five years had a relatively high severity. The research showed that assessments based on an MS severity score are applicable when the risk factors of suboptimal health are involved. By monitoring the scores over time, healthcare professionals can propose preventive strategies in time, thus enhancing the effectiveness of occupational health examination services.

## 1. Introduction

Metabolic syndrome (MS) is one of the risk factors for cardiovascular diseases (CVD) and Type 2 diabetes. MS not only leads to personal health issues but also burdens medical and health-care systems. The prevalence of MS has also increased in line with rising obesity rates and aging populations. A recent systematic review on the prevalence of MS among adults in the Asia-Pacific region found that MS had grown 30%, and in some cases even doubled, over the past decade [1]. Despite this growth, MS prevention can be achieved by assessing and ameliorating the relevant risk factors, including personal lifestyle habits and socio-economic conditions (occupation, educational level, and income) [2,3]. A study found that for patients with diabetes or CVD, the diagnosis of MS is redundant, and therefore the author recommended that patients already diagnosed with CVD and diabetes should be excluded from the diagnostic criteria for MS [4]. Moreover, the present classification for MS has yet to include age and the severity of indicators. Hence, with the aid of health education, the recommendations for personal disease prevention provided by physicians for different populations grouped by their risk of developing the aforementioned diseases can help promote and enhance their health [5,6,7]. A survey by the World Health Organization’s (WHO) experts pointed out that MS is not suitable for a clinical diagnosis and that other population-based prevention strategies should be developed and assessed [8]. Experts from the American Diabetes Association and the European Association for the Study of Diabetes have also jointly stated that there is an information gap in the current definition of MS. The definition fails to take into account age and other demographic differences and excludes other relevant risk factors. It also regards each of the five MS components as having equal degrees of impact on CVD or insulin resistance. It is therefore important for clinicians to assess separately and address the risk factors associated with CVD instead of just checking whether a patient has met the diagnostic criteria for MS. This statement highlighted the need to establish a full definition of MS that includes continuous variables in a multivariate scoring system [9]. Existing research relating to scoring a consistent MS measure has involved the use of various tools, including percentile ranking [10], Z-scores [11,12,13,14], principal component analysis [15,16], and confirmatory factor analysis [17,18,19].

In the past, most continuous MS scores were applied in studies of children and adolescents. Some studies used an MS Z-score to estimate the cutoff points for age-specific percentiles for men and women. The results indicated that this approach is beneficial for clinicians in assessing MS correlation between children and their parents. In some cases, this approach also allows for the development of informational applications equipped with calculator functions [13,14]. With regard to the application of continuous MS scores for adults, some authors who employed principal component analysis have found that adults with MS have a higher continuous MS score, which progressively increases with an increasing number of components [16]. Gurka et al. employed confirmatory factor analysis to assess the weighted contribution of MS components among different genders and racial groups. The authors found that the MS severity score developed by them could effectively assess the changes in a person’s risk of developing MS over time [17]. The same authors further released a cloud-based calculator online that directly calculates a person’s MS risk [18]. In addition to their Western counterparts, Asians researchers have also taken the initiative to develop MS severity scores [19,20]. These researches have also applied their MS severity scores on correlation analyses of various diseases, thus broadening the scientific application of these numerical indicators.

In contrast to the criteria for determining any abnormal values of MS components, numerical indicators of MS can facilitate one’s self-assessment of improving or deteriorating health conditions. This highlights the value of such indicators in preventive medicine. In Taiwan, workers in institutional organizations are required by law to undergo regular health examinations. However, their health reports enable them to check for abnormal values in single examination items and, hence, there is a lack of a comprehensive indicator for their CVD risk. The development of a numerical indicator for specific worker populations would allow health examination institutions to consolidate each worker’s numerous MS severity data and formulate a comprehensible graphic that serves as a reference for customizing health management strategies, as well as facilitating research pertaining to occupational risk factors. To the best of our knowledge, few studies have compared the MS risk of workers based on such numerical indicators. Therefore, this study performed a CVD risk assessment for worker populations by means of an MS severity score on the basis of the workers’ lifestyle habits and occupational characteristics. It also assessed the feasibility of applying and developing this numerical indicator in occupational health contexts.

## 2. Materials and Methods

### 2.1. Sources of Data

The data used in this study was collected from the health examination center of a regional teaching hospital in Taiwan. In 2018, workers from 10 large companies underwent their annual labor health examination at the center. Using a questionnaire, we collected the workers’ biochemistry examination items, which included the five components of MS: waist circumference, blood pressure, fasting plasma glucose, triglyceride level, and high-density lipoprotein (HDL) cholesterol. We also collected data relating to their sociodemographic characteristics: gender, date of birth, date of employment or job tenure, name of company, job title, lifestyle habits (hours of sleep, drinking, smoking, and betel nut chewing), and personal medical history. Because the data usage entailed various ethical issues, the protocol had to first receive approval from the Institutional Review Board of the said hospital (approval number IRB108B1101) where the research was conducted before the data were obtained and analyzed. A total of 7232 workers completed their health examinations. After removing 21 workers who were below 20 years and over 65 years of age and those with existing CVD, stroke, and diabetes (758 people), the remaining 6,453 subjects made up the sample size for this study.

### 2.2. Research Variables

The definition of MS in this study follows the criteria of the Health Promotion Administration of the Taiwan Ministry of Health and Welfare. A person is diagnosed with MS when three or more of the five MS components are within abnormal ranges. These five components are as follows: waist circumference ≥90 cm for men and ≥ 80 cm for women; fasting plasma glucose ≥ 100 mg/dL (5.55 mmol/L) or antidiabetic use; systolic blood pressure ≥ 130 mmHg, diastolic blood pressure ≥ 85 mmHg, or antihypertensive use; triglyceride level ≥ 150 mg/dL; and HDL cholesterol ≤ 40 mg/dL in men or ≤ 50 mg/dL in women. The mean and standard deviation of each of the five MS components were calculated based on the subjects’ gender and age group (20–34, 35–54, and 55–65 years). Then a determination was made as to whether the values were within an abnormal range, followed by an analysis of the distribution of the demographic characteristics. In terms of continuous indicators, we derived the MS severity score for each subject based on the five MS components according to each subject’s gender and age group. In particular, the inversed values of HDL cholesterol and the natural logarithms of triglyceride level were used. The standardized scores of the five MS components were then summed up to obtain the total MS severity score.

The risk factors in this study included a person’s lifestyle habits, which include drinking, hours of sleep, smoking, and betel nut chewing, as well as their occupational field, job title, and tenure. In the raw data, these variables and each subject’s medical history were recorded in natural language and must first undergo natural language processing prior to classification. This study employed the Jieba text segmentation tool, which is based on the Python programming language, for processing and classification. The classified predictive variables were then redefined according to type or order, such as smoking (yes/no), drinking (yes/no), betel nut chewing (yes/no), hours of sleep (≤6 h, 7 h, ≥8 h), occupational field (electronics/food/traditional industries/logistics), job title (technician/administrator/manager), and job tenure (<5 years, 5–10 years, 11–20 years, >20 years).

### 2.3. Statistical Analysis

This study adopted the method employed by Gurka et al. [17] by performing a confirmatory factor analysis and weighting of the MS components and a chi-square estimation based on factor loading equivalence. Model comparisons were performed based on the chi-square statistics and the Akaike information criterion (AIC). A smaller chi-square statistic and AIC results indicate a better model fit. The other fit indices included the root mean square error of approximation (RMSEA) and the standardized root mean square residual (SRMR). Here, a poor model fit was indicated by a RMSEA greater than 0.1 or a SERMR greater than 0.05. For the goodness of fit index (GFI), the normed fit index (NFI), and the Bentler–Bonett normed fit index, a poor model fit is indicated by a value smaller than 0.90. This study employed the SPSS Amos software (IBM corp., NY, USA) for confirmatory factor analysis in which a one-way path analysis was performed on the data and model fit. In order to realize the clinical interpretation and utilization of the results, during factor analysis, we first established equations based on the values of the MS components. The MS severity scores for gender and age-specific groups were then obtained through a covariance matrix by back-transforming the standardized scores derived from the aforementioned equations. A score close to 0 indicates that a person has an average risk of developing MS, while a higher or lower score indicates that a person has a higher- or lower-than-average risk.

In this study, the outcomes of the MS components were categorized as binary and continuous indicators, and they were respectively analyzed by means of binary logistic regression and multiple linear regression. For the logistic regression, the estimated risk was deduced using an adjusted odds ratio. For the linear regression, each variable was stratified and transformed into a dummy variable, and assessments of the increases or decreases in MS severity scores were performed by looking for the absence or presence of strata in each variable. This study employed SPSS for Windows Version 18 for regression analysis, with the level of significance set at 0.05. This study also employed the R statistical software to build the covariance matrix and to illustrate the distribution of the MS severity scores.

## 3. Results

**Figure 1:**
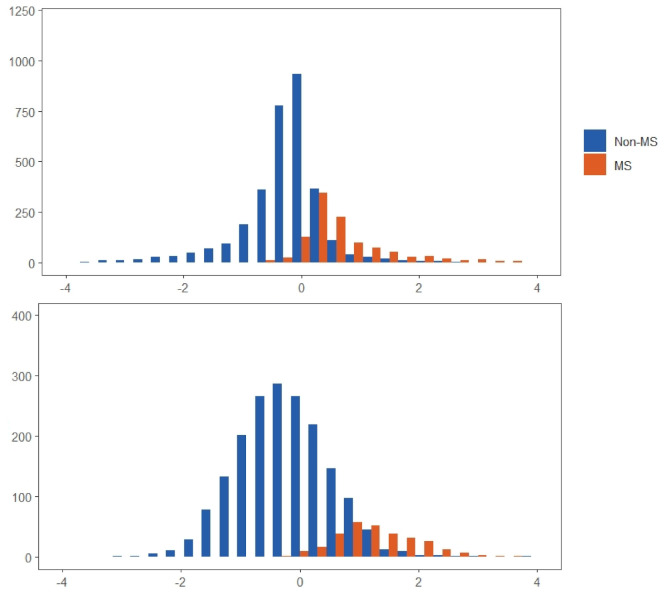
MS and MS severity score distribution for men (upper panel) and women (lower panel).

This study involved 4327 men (67.1%) and 2,126 women (32.9%) as the sample size distribution. By age group, there were 1852, 3241, and 1360 subjects (28.7%, 50.2%, and 21.1%, respectively) in the 20–34, 35–49, and 50–64 years age group, respectively (these values are not presented in a table). Table 1 shows that the prevalence rate of MS among men and women was 25.8% and 14.3%, respectively. Even though the prevalence was lower in women, it still differed from that in men, as there was a gradual increase in the prevalence among women older than 50 years. Figure 1 displays the distribution of MS and the MS severity scores in the sample. Regardless of gender, the subjects with MS mostly had a severity score that was higher than zero; however, among the subjects without MS, there were a notable proportion of them who had a severity score higher than zero, while very few had a severity score higher than two. This shows that a severity score between zero and two can be used to observe the coexistence of MS in a subject, in which men have a higher ratio of coexistence. Table 2 presents the factor loadings generated subjecting the MS components to a confirmatory factor analysis. There were differences between the factor loadings with respect to age and gender. Of the MS components, waist circumference had the highest factor loadings, which suggests that it contributed the most to MS risk and affected men to a greater extent. With the exception of women in the 50–64 age group, fasting plasma glucose had the lowest contribution. The factor loadings for HDL-cholesterol were highest in men above 35 years, whereas systolic blood pressure had a greater contribution in men below 35 years, while there were no overt age-specific differences in women. The MS severity score of each subject then was estimated based on an equation transformed from factor loadings of the five MS components.

Table 3 provides a breakdown of the subjects’ MS severity scores and prevalence based on their demographic characteristics, occupations, and lifestyle habits. In terms of gender and age, the prevalence of MS in men was almost double that in women, while it was about 10% lower in subjects aged below 35 years than subjects aged above 35 years. In terms of occupation, the prevalence was about 25% in workers in the electronics and food industries, which was slightly higher than those in traditional industries, but was higher by 8% than those in the logistics industry. The prevalence was higher by 10% among managers compared to technicians, and this figure was almost double of that of administrators. Workers with a job tenure of more than five years had an MS prevalence that exceeded 22%, which was markedly higher than that of workers with a job tenure of less than five years (15.4%). Those who smoke, chew betel nut, and drink had a 9%, 15%, and 2.5% higher prevalence, respectively, than those who did not have any of these habits. Compared to those who slept more than eight hours a day, the prevalence of MS was 3% and 6% higher among those who slept between six to seven hours and less than six hours per day, respectively. The distributions of the MS severity scores were also similar. However, given that the scores have been stratified by age and gender, the age- and gender-specific demographic variables had no reference values. Nevertheless, it can be seen that workers in the food or electronics industries, working as technicians or managers, with a job tenure from five to 20 years, with smoking or betel nut chewing habits or slept less than six hours per day had positive MS severity scores. In other words, these workers have a higher propensity of developing more severe MS.

Table 4 shows the estimated risk factors of MS in two regression models. After adjusting for gender and age, workers in the traditional, food, and electronics industries had a 30%, 80%, and 70% increased risk of MS, respectively. Even though managers had a 34% increased risk of MS compared to administrators, the difference was not significant. Those with a job tenure of more than five years had a 30% to 40% increased risk of MS compared to those working for less than five years. In terms of lifestyle habits, those who smoke, chew betel nut, or slept less than six hours per day had a significantly higher (20% to 50%) risk of MS. In contrast, those who drink had a relatively lower risk. With regard to MS severity scores, the scores of workers in the traditional, food, and electronics industries were higher by 0.11 to 0.33 compared to those in the logistics industry. The severity score of managers was significantly higher by 0.14 than that of administrators. Those with a job tenure of more than five years had relatively high severity score (ranging from 0.09 to 0.14) than those working for less than five years. In terms of lifestyle habits, those who chew betel nut had a 0.17 higher score than those who do not. Those who slept less than six hours per day had a 0.07 higher score than those who slept more than eight hours per day. Finally, those who drink had a 0.07 lower score than those who do not.

## 4. Discussion

According to the factor analysis models, waist circumference was found to have the highest influence on MS among the five components, and the degree of influence was higher among men as compared to women. HDL-cholesterol and systolic pressure had a greater influence on middle-aged workers and younger workers, respectively. A study on healthy aging in the human lifespan highlighted that waist circumference had the highest predictive power for MS among young adults [21]. Yu et al. suggested that the predictive power of waist circumference for MS was higher in men than in women [22]. Another cohort study on ethnic Chinese populations showed that after adjusting for age, smoking habits, drinking habits, exercise habits, and education level, waist circumference had the highest predictive power for MS [23]. The five components of MS have different health implications for different populations. Therefore, health managers should develop specific improvement strategies based on the demographic differences among people with MS. The equations for estimating MS severity in different age groups had acceptable model fits, and hence could serve as indicators for labor health examination agencies to follow up on the health statuses of labors and to develop relevant health management plans.

This study also found that the prevalence of MS differed in men and women. Common lifestyle-related risk factors that could increase the risk of MS and its severity include smoking, betel nut chewing, and lack of sleep. The utilization of different indicators and models could result in minor differences between the validation results of the risk factors. In terms of occupational field, the risk of MS was higher for workers in the electronics and food industries, and those with a job tenure of less than five years were at a lower risk of MS. The dichotomized model showed no differences between the risk of MS in terms of workers’ job title, whereas the severity score model indicated that managers had a higher risk of MS. In general, the influence of lifestyle-related risk factors on MS was consistent with the findings of a majority of past studies pertaining to health promotion. Yet, there is room for further discussions of occupation-related risk factors. A cohort study showed that the prevalence of MS was around 19% among male Korean workers. In particular, heavy drinkers, non-office workers, and vehicle drivers had higher risks [24]. Another nationwide cross-sectional study on Korean workers revealed that 24% and 20% of men and women, respectively, had MS and that MS was less prevalent among male manual workers compared to their non-manual counterparts [3]. Even though this study found that MS prevailed more in technicians than administrators, the difference was not significant after adjusting for occupational field (e.g., electrics and food industry) and other variables. This suggests that the differences among job titles could have be diminished by other factors such as occupational field. The aforementioned Korean study [3] also suggested that the lower prevalence of MS among technicians could be attributed to their higher education level and socioeconomic status. In this present study, however, it was found that managers have higher health risks. Since most of the companies examined in this study are from technical industries, these managers may have been promoted from technical departments. If so, it is worth examining whether certain causes other than socioeconomic status created theoretical differences. An African study found that, despite having higher education levels and physical activity levels, the prevalence of MS was higher among technicians than schoolteachers. The authors hypothesized that this may be associated with the fact that technicians have a higher occupational exposure to dust, oil, and organic and inorganic substances [25]. Another study on Latino workers deduced that occupational exposure to organic solvents may increase the risk of hypertension. The same study also pointed out that large sample sizes increase the difficulty of assessing a person’s occupational exposure to chemicals in reality, which could very well limit the accuracy of the research findings [26].

In a recent study, Kim and Yun pointed out that male day workers are at a higher risk of MS compared to male shift workers. The prevalence of MS among women was expressed in terms of sleep duration: women who slept more than eight hours per day had a higher risk of MS than those who slept less than six hours [27]. The results of these sleep-related risk factors in the cited studies differed with those in this study. Since the present study also employed the cross-sectional approach for estimation, the causal relationship between sleep duration and health remains unconfirmed. A study concluded that the prevalence of MS among American workers was 21%, in which food industry workers and farm operators had the highest prevalence (up to 30%). Engineers and scientists had a lower prevalence (9%). After adjusting for various factors, transportation and logistics workers had a higher risk of MS relative to managers. The authors suggested that the high prevalence of MS among these workers could be associated with irregular working hours, rotating shifts, job stress, and sleep problems [28]. In this present study, most of the logistics workers have fixed shifts and so their lower risk of MS could be due to the lower number of rotating shifts or irregular working hours relative to other occupations.

After comparing the dichotomized and numerical models of MS indicators, this study found that, in certain factors such as job title at least, assessing MS components through the numerical indicators is more sensitive than through the dichotomized indicators. With regard to MS scoring, the Framingham risk score (FRS) can be used to predict a person’s risk of CVD for the next 10 years. Nevertheless, the FRS does not take into account obesity and plasma glucose, which limits of its potential as a tool for making disease prediction and developing prevention strategies for different age groups [29,30]. Previous studies on Asian populations also revealed that the risks assessed through the FRS could be underestimated [2,31]. Consequently, people perceived to have lower risks according to the FRS could easily be overlooked by physicians, thereby reducing their chances for early prevention. Eisenmann suggested the use of an MS score to estimate the mortality risk of type 2 diabetes, atherosclerosis, and CVD, as this is necessary for clinicians to perform treatments for patients during their disease. However, the researcher also argued that an MS score derived from a specific population cannot be inferred to other populations [12]. To this end, an MS score should differ according to different populations, gender, and age distributions. With regard to Asian populations, Kang et al. studied Chinese subjects and found that an MS score had a higher accuracy for predicting CVD risk compared to dichotomized criteria and recommended that appropriate scoring systems should be developed for different racial groups and regions [32]. After collecting data of a general population from a large health screening center, a recent Taiwanese study revealed strong correlations between MS severity scores and lifestyle habits such as physical activity, drinking alcohol, consuming sweetened beverages, and smoking, which was a finding that was inconsistent with traditional MS diagnosis [33]. From the perspective of occupational health, the present study estimated the MS severity among different groups of workers and found that this approach has discriminatory power in terms of health risk assessments in the workplace. As such, we recommend that this approach can be applied in the field of occupational health risk assessment as it offers a reference for subsequent clinical practice or research.

Nonetheless, the applicability of the research findings is subjected to some limitations. The cross-sectional study design may restrict the establishment of causal hypotheses. Additionally, since the subjects of this study were workers who undertook their health examinations at a specific healthcare institution, the MS severity scores cannot be applied to workers from other workplaces. Next, due to the lack of data related to lifestyle habits and behaviors, such as exercise habits, diets, and occupational exposure, the completeness of the findings with respect to risk assessments could be reduced. Furthermore, this study aimed to assess occupational risk in an early clinical context. Patients with CVD were excluded and some missing raw data resulted in the exclusion of medication use, therefore, the present MS severity score can only be applied to workers without CVD. It is recommended that weights should be allocated to variables such as personal disease or medication use in future studies, with the purpose of developing an MS severity score that is applicable to all types of workers. Finally, the effects of selected participants will limit our findings to generalize to a different population. Further analyses with data from other source are need.

## 5. Conclusions

The objective of developing an MS severity score in the present study is not to replace the discriminatory function of traditional MS assessment approaches, but to examine the feasibility of employing the numerical value as an indicator of the deterioration or improvement in workplace health risks. Conclusively, the research findings showed that assessments or predictions based on an MS severity score are applicable when the risk factors of suboptimal health are involved. In the future, health examination institutions can develop informative modules related to health examinations and examination items that enable customized and automated estimations in a quick and simple manner. By monitoring the changes in workers’ health examination scores over time on a regular basis, healthcare professionals can propose preventive strategies in a timely manner for people at risk of underlying MS, thus enhancing the effectiveness of these health examination services.

## Figures and Tables

**Table 1:** The distribution of MS and the five MS components among men and women.

Demography	*n*	MS (%)	Waist Circumference (cm)		Fasting Plasma Glucose (mg/dL)		Triglycerides(mg/dL)		High-Density Lipoprotein (mg/dL)		Systolic Blood Pressure (mmHg)	
			Mean	Sd	Mean	Sd	Mean	Mean	Sd	Mean	Sd	Mean
Men	4327	25.8	84.45	10.43	98.49	22.21	142.07	138.37	49.89	11.59	130.08	14.57
20–34 years	1355	16.2	82.5	11.32	93.11	12.57	117.91	84.53	50.69	11.04	127.87	12.84
35–49 years	2145	30.3	85.79	10.32	99.66	23.67	155.37	156.71	49.03	11.74	130.67	14.69
50–64 years	827	29.9	84.2	8.45	104.28	28.03	147.19	152.74	50.81	11.91	132.17	16.35
Women	2126	14.3	75.38	10.24	94.91	17.05	91.06	52.03	60.7	13.57	124.28	16.34
20–34 years	497	9.9	73.3	11.49	90.71	18.48	81.01	53.62	60.62	14.36	119.94	13.31
35–49 years	1096	14.3	76.16	10.33	95.05	15.96	90.32	50.54	59.87	13.31	123.36	16.02
50–64 years	533	18.6	75.71	8.4	98.54	16.94	101.96	51.46	62.49	13.17	130.21	17.83

Abbreviations: MS = metabolic syndrome; Sd = standard deviation.

**Table 2:** Factor loadings generated through the confirmatory factor analysis of the MS components.

MS Components	Men			Women		
	20–34 Years	35–49 Years	20–34 Years	35–49 Years	20–34 Years	35–49 Years
Waist circumference	0.82	0.75	0.75	0.78	0.71	0.62
Fasting plasma glucose	0.25	0.26	0.24	0.31	0.34	0.42
Ln-Triglycerides	0.41	0.48	0.51	0.50	0.52	0.41
High-density lipoprotein	0.34	0.45	0.48	0.36	0.45	0.40
Systolic blood pressure	0.48	0.34	0.30	0.51	0.46	0.49

**Table 3:** Prevalence and severity score of MS among subjects with different demographic characteristics, occupations, and lifestyle habits.

	MS		MS Severity Score	Total
	*n*	%	Mean (Sd)	
Sex				
Male	1116	25.8	–0.004 (1.000)	4327
Female	305	14.4	–0.005 (1.000)	2126
Age (years)				
20–34	268	14.5	–0.012 (1.000)	1852
35–54	1008	25.2	–0.001 (0.994)	4002
55–64	145	24.2	–0.006 (1.039)	599
Occupational field				
Electronics	525	24. 8	0.164 (1.001)	2118
Food	110	24.6	0.019 (1.123)	448
Traditional industries	535	22.5	–0.057 (0.961)	2377
Logistics	251	16.6	–0.165 (0.984)	1510
Job title				
Technician	1122	22.9	0.012 (0.994)	4902
Administrator	241	17.5	–0.083 (1.027)	1377
Manager	58	33.3	0.150 (0.914)	174
Job tenure (years)				
<5	288	15.4	–0.075 (0.934)	1872
5–10	259	22.2	0.082 (1.128)	1169
11–20	409	27.0	0.043 (1.022)	1516
>20	463	24.5	–0.026 (0.955)	1890
Missing	2	33.3	–0.006 (0.581)	6
Smoke				
No	946	19.8	-0.026 (0.985)	4789
Yes	475	28.5	0.058 (1.038)	1664
Drink				
No	806	21.0	0.009 (1.000)	3840
Yes	615	23.5	–0.024 (0.999)	2613
Betel chewing				
No	1253	21.0	–0.017 (0.995)	5967
Yes	168	34.6	0.149 (1.046)	486
Sleep(h/day)				
≤6	462	25.0	0.050 (1.023)	1850
7	694	21.9	–0.016 (1.007)	3175
≥8	264	18.6	–0.049 (0.950)	1421
Missing	1	14.3	–0.033 (0.224)	7

**Table 4:** The estimated risk factors of MS in two regression models.

	Logistic Regression		Linear Regression	
	AOR	95%CI	β	95%CI
Occupational field (vs. Logistics)				
Electronics	1.722	1.428–2.077	0.328	0.256–0.399
Food	1.835	1.405–2.396	0.190	0.083–0.297
Traditional industry	1.323	1.106–1.581	0.109	0.041–0.177
Job title (vs. Administrator)				
Technician	0.982	0.826–1.168	–0.010	–0.077–0.057
Manager	1.341	0.933–1.926	0.135	0.025–0.296
Job tenure (vs. <5 years)				
5–10 years	1.320	1.081–1.612	0.138	0.063–0.214
11–20 years	1.410	1.143–1.739	0.102	0.018–0.185
>0 years	1.307	1.056–1.618	0.094	0.010–0.178
Smoke (vs. No)				
Yes	1.232	1.058–1.434	0.058	–0.006–0.122
Drink (vs. No)				
Yes	0.862	0.755–0.985	–0.067	–0.12– –0.013
Betel chewing (vs. No)				
Yes	1.533	1.224–1.919	0.167	0.066–0.269
Sleep (vs. ≥8 h/day)				
≤6 h/day	1.345	1.127–1.605	0.071	0.001–0.140
7 h/day	1.198	1.017–1.411	0.027	–0.036–0.089

The calculation of the adjusted odds ratios (AOR) and β was performed using logistic and linear regression models that were adjusted with all covariates, which include demographic variables.

## References

[1] Ranasinghe R., Mathangasinghe Y., Jayawardena R., Hills A.P., Misra A. (2017). Prevalence and trends of metabolic syndrome among adults in the Asia-Pacific region: A systematic review. BMC Public Health.

[2] Liao C.M., Lin C.M. (2018). Life course effects of socioeconomic and lifestyle factors on metabolic syndrome and 10-year risk of cardiovascular disease: A longitudinal study in Taiwan adults. Int. J. Environ. Res. Public Health.

[3] Ryu J.Y., Hong S., Kim C.H., Lee S., Kim J.H., Lee J.T., Kim D.H. (2013). Prevalence of the metabolic syndrome among Korean workers by occupational group: Fifth Korean National Health and Nutrition Examination Survey (KNHANES) 2010. Ann. Occup. Environ. Med..

[4] Gale E.A.M. (2008). Should we dump the metabolic syndrome? Yes. BMJ.

[5] Kastelein J. (2004). Cardiovascular risk-through the ages. Atheroscler. Suppl..

[6] Tuomilehto J. (2004). Impact of age on cardiovascular risk: Implications for cardiovascular disease management. Atheroscler. Suppl..

[7] Sattar N., Forouhi N.G. (2005). Metabolic syndrome criteria: Ready for clinical prime time or work in progress?. Eur. Heart J..

[8] Simmons R.K., Alberti K.G., Gale E.A., Tuomilehto J., Qiao Q., Ramachandran A., Tajima N., Brajkovich Mirchov I., Ben-Nakhi A., Reaven G. (2010). The metabolic syndrome: Useful concept or clinical tool? Report of a WHO Expert Consultation. Diabetologia.

[9] Kahn R., Buse J., Ferrannini E., Stern M. (2005). The metabolic syndrome: Time for a critical appraisal: Joint statement from the American Diabetes Association and the European Association for the Study of Diabetes. Diabetes Care.

[10] Raitakari O.T., Porkka K.V., Rasanen L., Ronnemaa T., Viikari J.S. (1994). Clustering and six year cluster-tracking of serum total cholesterol, HDL-cholesterol and diastolic blood pressure in children and young adults. The Cardiovascular Risk in Young Finns Study. J. Clin. Epidemiol..

[11] Eisenmann J.C., Wickel E.E., Welk G., Blair S.N. (2007). Combined influence of cardiorespiratory fitness and body mass index on cardiovascular disease risk factors among 8-18 year old youth: The Aerobics Center Longitudinal Study. Int. J. Pediatr. Obes..

[12] Andersen L.B., Harro M., Sardinha L.B. (2006). Physical activity and clustered cardiovascular risk in children: A cross-sectional study (The European Youth Heart Study). Lancet.

[13] Guseman E.H., Eisenmann J.C., Laurson K.R., Cook S.R., Stratbucker W. (2018). Calculating a continuous metabolic syndrome score using nationally representative reference values. Acad. Pediatr..

[14] Cook S., Auinger P., Huang T.T.K. (2009). Growth curves for cardio-metabolic risk factors in children and adolescents. J. Pediatr..

[15] Katzmarzyk P.T., Perusse L., Malina R.M., Bergeron J., Despres J., Bouchard C. (2001). Stability of indicators of the metabolic syndrome from childhood and adolescence to young adulthood: The Quebec Family Study. J. Clin. Epidemiol..

[16] Wijndaele K., Beunen G., Duvigneaud N., Matton L., Duquet W., Thomis M., Lefevre J., Philippaerts R.M. (2006). A Continuous Metabolic Syndrome Risk Score: Utility for epidemiological analyses. Diabetes Care.

[17] Gurka M.J., Lilly C., Oliver M.N., DeBoer M.D. (2014). An examination of sex and racial/ethnic differences in the metabolic syndrome among adults: A confirmatory factor analysis and a resulting continuous severity score. Metabolism.

[18] Gurka M.J., DeBoer M.D., Filipp S.L. (2019). MetS Calc: Metabolic syndrome severity calculator. Cardiovasc. Diabetol..

[19] Huh H., Lee J.H., Moon J.S., Sung K.C., Kim J.Y., Kang D.R. (2019). Metabolic syndrome severity score in Korean adults: Analysis of the 2010–2015 Korea National Health and Nutrition Examination Survey. J. Korean Med. Sci..

[20] Low S., Khoo K.C.J., Wang J., Irwan B., Sum C.F., Subramaniam T., Lim S.C., Wong T.K.M. (2019). Development of a metabolic syndrome severity score and its association with incident diabetes in an Asian population—Results from a longitudinal cohort in Singapore. Endocrine.

[21] Beydoun M.A., Kuczmarski M.T., Wang Y., Mason M.A., Evans M.K., Zonderman A.B. (2011). Receiver-operating characteristics of adiposity for metabolic syndrome: The healthy aging in neighborhoods of diversity across the life span (HANDLS) study. Public Health Nutr..

[22] Yu J., Tao Y., Tao Y., Yang S., Yu Y., Li B., Jin L. (2016). Optimal cut-off of obesity indices to predict cardiovascular disease risk factors and metabolic syndrome among adults in Northeast China. BMC Public Health.

[23] Liu L., Liu Y., Sun X., Yin Z., Li H., Deng K., Chen X., Cheng C., Luo X., Zhang M. (2018). Identification of an obesity index for predicting metabolic syndrome by gender: the rural Chinese cohort study. BMC Endocr. Disord..

[24] Kang S.H., Hwang S.Y. (2016). Influence of Occupational Type and Lifestyle Risk Factors on Prevalence of Metabolic Syndrome among Male Workers: A Retrospective Cohort Study. Korean J. Adult Nurs..

[25] Adeseye A., Oloyede T.W. (2017). Metabolic syndrome and occupation: Any association? Prevalence among auto technicians and school teachers in South West Nigeria. Diabetes Metab. Syndr. Clin. Res. Rev..

[26] Bulka C.M., Daviglus M.L., Persky V.W., Durazo-Arvizu R.A., Avilés-Santa M.L., Gallo L.C., Hosgood H.D., Singer R.H., Talavera G.A., Thyagarajan B. (2017). Occupational exposures and metabolic syndrome among hispanics/latinos. J. Occup. Environ. Med..

[27] Kim K.-Y., Yun J.-M. (2019). Analysis of the association between health-related and work-related factors among workers and metabolic syndrome using data from the Korean National Health and Nutrition Examination Survey (2016). Nutr. Res. Pract..

[28] Davila E.P., Florez H., Fleming L.E., Lee D.J., Goodman E., Leblanc W.G., Caban-Martinez A.J., Arheart K.L., McCollister K.E., Christ S.L. (2010). Prevalence of the metabolic syndrome among US workers. Diabetes Care.

[29] Berry J.D., Lloyd-Jones D.M., Garside D.B., Greenland P. (2007). Framingham risk score and prediction of coronary heart disease death in young men. Am. Heart J..

[30] Hemann B.A., Bimson W.F., Taylor A.J. (2007). The Framingham Risk Score: An Appraisal of Its Benefits and Limitations. Am. Heart Hosp. J..

[31] Khanna R., Kapoor A., Kumar S., Tewari S., Garg N., Goel P.K. (2013). Metabolic syndrome & Framingham Risk Score: Observations from a coronary angiographic study in Indian patients. Indian J. Med Res..

[32] Kang G.-D., Guo L., Guo Z., Hu X.-S., Wu M., Yang H.-T. (2012). Continuous metabolic syndrome risk score for predicting cardiovascular disease in the Chinese population. Asia Pac. J. Clin. Nutr..

[33] Lin C.-M. (2020). An application of metabolic syndrome severity scores in the lifestyle risk assessment of taiwanese adults. Int. J. Environ. Res. Public Health.

